# Delayed tumour enhancement on gadoxetate-enhanced MRI is associated with overall survival in patients with colorectal liver metastases

**DOI:** 10.1007/s00330-018-5618-5

**Published:** 2018-07-10

**Authors:** Helen M. C. Cheung, Paul J. Karanicolas, Natalie Coburn, Vikrum Seth, Calvin Law, Laurent Milot

**Affiliations:** 1 Department of Medical Imaging, Sunnybrook Health Sciences Centre, University of Toronto, 2075 Bayview Avenue, Rm AB 202, Toronto, ON M4N 3M5 Canada; 2 Department of Surgery, Sunnybrook Health Sciences Centre, University of Toronto, 2075 Bayview Avenue, Toronto, ON M4N 3M5 Canada

**Keywords:** Colorectal cancer, Neoplasm, Metastases, Gadolinium, Survival

## Abstract

**Objectives:**

To determine whether tumour enhancement on preoperative delayed-phase gadoxetate-enhanced MRI can predict long-term survival in patients with colorectal liver metastases (CRCLM) post-hepatectomy.

**Materials and methods:**

Sixty-five patients who received a preoperative gadoxetate-enhanced MRI prior to liver resection for CRCLM from January 1, 2010, to December 31, 2012, were included in this retrospective study. Target tumour enhancement (TuEn) was calculated as the mean percentage increase in SNR from precontrast to 10-min or 20-min delayed phase for up to two target lesions. Per-patient TuEn was stratified into weak and strong enhancement based on the cut-off determined by the Youden Index for 3-year survival. Kaplan-Meier and Cox regression analyses were used to determine whether tumour enhancement could predict overall survival independent of potential confounders (clinical risk score).

**Results:**

The proportion surviving at 3 years was 85.1% in patients with strong TuEn at 10 min vs. 56.5% in those with weak TuEn at 10 min (*p* = 0.001). The proportion surviving at 3 years was 79.4% in patients with strong TuEn at 20 min vs. 58.7% in those with weak TuEn at 20 min (*p* = 0.011). After adjusting for potential confounders, the hazard ratio of death was 0.24 (*p* = 0.009) in patients who had weak TuEn at 10 min and 0.32 (*p* = 0.018) in patients who had weak TuEn at 20 min.

**Conclusions:**

Strong delayed tumour enhancement seen on gadoxetate-enhanced MRI is associated with overall survival in patients with CRCLM post-hepatectomy and may be useful for preoperative risk stratification.

**Key Points:**

• *Delayed tumour enhancement of colorectal liver metastases on gadoxetate-enhanced MRI is associated with survival post-hepatectomy*

• *Delayed tumour enhancement of colorectal liver metastases on gadoxetate-enhanced MRI can be measured at both 10 min and 20 min post-contrast injection*.

## Introduction

Colorectal cancer is the third leading cause of cancer deaths worldwide (after lung and liver) [1]. Most of the deaths are related to metastatic disease, with the liver being the most common site for metastases. With improvements in surgical techniques and systemic therapy in the last 2 decades, the survival for colorectal liver metastases (CRCLM) has significantly improved. In patients who are candidates for surgery, 5-year survival rates are in the range of 25-40% [2, 3].

It has recently been shown that late gadolinium enhancement of CRCLM on preoperative magnetic resonance imaging (MRI) with an extracellular contrast agent, gadobutrol, is associated with tumour fibrosis and overall survival post-hepatectomy [4]. Late gadolinium enhancement of CRCLM on MRI with gadobutrol may be associated with tumour fibrosis due to leakage of extracellular contrast into the tumour via the interstitium [4]. It has previously been established in the histology literature that tumour fibrosis is associated with good outcomes post-hepatectomy [5, 6].

Although MRI with extracellular contrast agents is still commonly used in many institutions, others now use MRI with hepatobiliary-specific contrast agents, such as gadoxetate (gadoxetic acid), to stage CRCLM [7]. Gadoxetate is known to have a dual mechanism: in addition to hepatobiliary-specific uptake, gadoxetate also has an extracellular component. Therefore, we would expect that CRCLMs would also demonstrate delayed enhancement on gadoxetate-enhanced MRI via its extracellular component. In fact, several previous papers have described late gadolinium enhancement of CRCLMs on delayed phase imaging [8, 9].

Although there is currently no literature on the timing of delayed phase imaging and correlation with tumour fibrosis in CRCLM, prior studies in the cardiac literature demonstrated that late gadolinium enhancement is best correlated with tissue fibrosis between 10-30 min using extracellular contrast agents [10]. Given that delayed phase imaging on routine liver MRI with gadoxetate is typically performed at 10 min and 20 min post-contrast, measurements of late gadolinium enhancement were performed at both of these time points [7].

Therefore, the goal of this study was to determine whether tumour enhancement of CRCLM seen on preoperative gadoxetate-enhanced MRI is associated with long-term survival post-hepatectomy at 10 min and 20 min post-contrast injection.

## Materials and methods

This retrospective study was approved by the institutional research ethics board, which waived the requirement for informed consent. Patients with CRCLM who received a preoperative MRI with gadoxetate (Primovist™, Eovist™) prior to hepatectomy from January 1, 2010, to December 31, 2012, at a single tertiary cancer centre were included in the study. Patients were excluded if they received portal vein embolisation prior to the MRI or if images were of unacceptable quality for analysis.

MRIs were obtained for clinical purposes (diagnosis or staging and/or preoperative planning). As per institutional clinical guidelines for liver MR imaging, patients received 3D axial T1 imaging (TE ~ 1.5 ms, TR ~ 3.0 ms, flip angle ~ 10 degrees) on the noncontrast, arterial, portovenous, 2-min, 5-min, 10-min, and 20-min delayed phases (Table 1). All patients received a 10-ml intravenous dose of gadoxetate at 1.0 mmol/ml. Studies were performed on 1.5- (GE Twinspeed™) or 3.0-T (Philips Achieva™) magnets with an eight-channel body phased array coil covering the entire liver. If a patient received multiple gadoxetate-enhanced MRIs prior to surgery, then the MRI performed closest to the date of surgery was used. We excluded patients who had their most recent MRI more than 9 months prior to surgery.

**Table 1:** MRI parameters for gadoxetate-enhanced MRI used in the study

MRI parameters	
Plane	Axial
TE	1.5 ms
TR	3.0 ms
Flip angle	10 degrees
Slice thickness	5 mm
Spacing	2.5 mm
Matrix	320 × 192
Field of view	360 mm
Contrast	Gadoxetate (10 ml of 1.0 mmol/ml iv)

Demographic and clinical information was obtained on age, sex, number of liver metastases, size of the liver metastases, time from diagnosis of primary to diagnosis of liver metastases, node positivity of the primary colorectal cancer, and preoperative carcinoembryonic antigen (CEA) level within 6 months prior to surgery. The primary outcome of the study was overall survival post-MRI. We excluded patients who did not receive preoperative chemotherapy. We also excluded patients who died within 90 days of surgery to exclude deaths related to surgery.

The commonly used, validated, five-point, preoperative clinical risk score developed by Fong et al was calculated [11]. This score allots one point for each of five prognostic variables (number of liver metastases, size of the liver metastases, time from diagnosis of primary to diagnosis of liver metastases, node positivity of the primary colorectal cancer, and preoperative CEA level within 6 months prior to surgery) [11].

All clinical information was obtained using a prospectively maintained surgical database. Updated mortality data were obtained using electronic patient charts and publicly available obituary databases.

### Imaging analysis

All imaging analysis was performed on the standard clinical picture archiving and communication system (PACS) software used at our institution (Agfa Impax 6.3.1, AGFA HealthCare N.V., Belgium1™). CRCLMs were identified, and the signal-to-noise ratio (SNR) of each metastasis on noncontrast and 10- and 20-min delayed phases was calculated using previously described methods according to the following formula:

1 $$ SNR=\frac{Signal\ intensity\ (tumour)}{Standard\ deviation\ (noise)} $$

Measurements of tumour signal intensity were performed at the axial level where the tumour was largest and by placing a round region of interest (ROI) most closely approximating the entire tumour. The background noise was calculated by taking eight 1-2-cm ROI samples surrounding the outside of the patient, but taking care to exclude banding due to motion artefact [4]:

The tumour enhancement at 10-min delayed phase (TuEn10) and 20-min delayed phase (TuEn20) for each CRCLM was calculated as the percentage increase in the SNR between the noncontrast and the 10-min and 20-min delayed phases, according to the following formula:

2 $$ TuEnX=\frac{ SNR X\hbox{--} SNR0}{SNR0}\ x\ 100\% $$

where SNRX is the signal-to-noise ratio of the liver metastasis at either 10- or 20-min delayed phase and SNR0 is the signal-to-noise ratio of the liver metastasis on noncontrast imaging.

If there were multiple lesions, then the two largest lesions were taken as target lesions and the mean TuEnX of the target lesion was taken as the target TuEnX, as described previously in the literature [4]. The mean TuEnX was used for survival analysis.

CRCLMs < 5 mm were excluded from analysis as they could not be accurately measured. Lesions were confirmed to be CRCLMs based on postoperative pathology reports.

### Survival analysis for target TuEnX

The Youden Index was used to determine the value of Target TuEn10, which has the best sensitivity and specificity for predicting 3-year overall survival [12]. Target TuEn10 was dichotomised into strong and weak enhancement using the cut-off obtained from the Youden Index, which was used for survival analysis.

A chi-square test was used to determine if there were differences in demographic data between the strong and weak Target TuEn10 groups (Table 2).

**Table 2:** Baseline demographics for patients with strong vs. weak tumour enhancement of colorectal liver metastases on delayed phase gadoxetate-enhanced MRI

Description	Weak target TuEn10 (*n* = 29)	Strong target TuEn10 (*n* = 36)	*p* value	Weak target TuEn20 (*n* = 22)	Strong target TuEn20 (*n* = 53)	*p* value
Age, years						
< 65	15 (51.7%)	21 (58.3%)	*p* = 0.59	11 (55.0%)	25 (56.8%)	*p* = 0.89
≥ 65	14 (48.3%)	15 (41.7%)		9 (45.0%)	19 (43.2%)	
Sex						
Male	12 (41.4%)	21 (58.3%)	*p* = 0.17	9 (45.0%)	23 (52.3%)	*p* = 0.59
Female	17 (58.6%)	15 (41.7%)		11 (55.0%)	21 (47.7%)	
Number of tumours						
# of tumours = 1	8 (27.6%)	13 (36.1%)	*p* = 0.47	6 (30.0%)	15 (34.1%)	*p* = 0.75
# of tumours > 1	21 72.4%)	23 (63.9%)		14 (70.0%)	29 (65.9%)	
Size of tumour						
Size ≤ 5cm	22 (75.9%)	34 (94.4%)	*p* = 0.03*	17 (85.0%)	38 (86.4%)	*p* = 0.88
Size > 5cm	7 (24.1%)	2 (5.6%)		3 (15.0%)	6 (13.6%)	
Time from diagnosis of primary to diagnosis of liver metastases						
Time < 12 months	11 (37.9%)	4 (11.1%)	*p* = 0.01*	5 (25.0%)	10 (22.7%)	*p* = 0.84
Time ≥ 12 months	18 (62.1%)	32 (88.9%)		15 (75.0%)	34 (77.3%)	
Node positive primary						
# of nodes positive < 5	25 (86.2%)	28 (77.8%)	*p* = 0.38	17 (85.0%)	35 (79.5%)	*p* = 0.60
# of nodes positive ≥ 5	4 (13.8%)	8 (22.2%)		3 (15.0%)	9c(20.5%)	
Preoperative carcinoembryonic antigen (CEA)						
CEA ≤ 200 ng/ml	25 (89.3%)	34 (94.4%)	*p* = 0.45	17 (85.0%)	41 (95.3%)	*p* = 0.16
CEA > 200 ng/ml	3 (10.7%)	2 (5.6 %)		3 (15.0%)	2 (4.7%)	
Data not available	1					
Clinical risk score						
< 3	19 (70.4%)	30 (83.3%)	*p* = 0.22	14 (77.8%)	34 (77.3%)	*p* = 0.97
≥ 3	8 (29.6%)	6 (16.7%)		4 (22.2%)	10 (22.7%)	
Data not available	2					
Magnet						
1.5 T	2 (6.9%)	8 (22.9%)	*p* = 0.08	3 (15.0%)	7 (15.9%)	*p* = 0.93
3.0 T	27 (93.1%)	27 (77.1%)		17 (85.0%)	37 (84.1%)	

*p < 0.05

Kaplan-Meier statistics were used to determine the univariate association between strong vs. weak Target TuEn10. Cox regression statistics were used to determine the multivariable association between strong vs. weak Target TuEn10 and overall survival after adjusting for clinical risk score.

Post-hoc sensitivity analyses were performed using Cox regression for any demographic variables that demonstrated significant differences between strong vs. weak Target TuEn10 (Table 2).

The analysis was repeated for Target TuEn20.

All analyses were performed on SPSS (IBM SPSS Statistics for Macintosh, version 22.0, 2013. Armonk, NY: IBM Corp.).

## Results

### Patient population

Sixty-five patients met inclusion/exclusion criteria for this study. Of 100 patients who had a preoperative gadoxetate-enhanced MRI prior to surgery for CRCLM, we excluded 8 where imaging was unavailable for analysis, 3 because of poor quality imaging, 4 who had portal vein embolisation prior to MRI, 2 who had undergone MRI more than 9 months prior to surgery, 11 who did not receive preoperative chemotherapy, and 7 patients who died within 90 days of surgery.

The average age of study patients was 61.5 years (SD: 12.4 years). There were 33 males (50.8%) and 32 females (49.2%). Baseline demographics are shown in Table 2. The time from MRI to surgery ranged from 0-9 months with a median time of 2.0 months.

### Survival analysis for target TuEn10

**Fig. 1:**
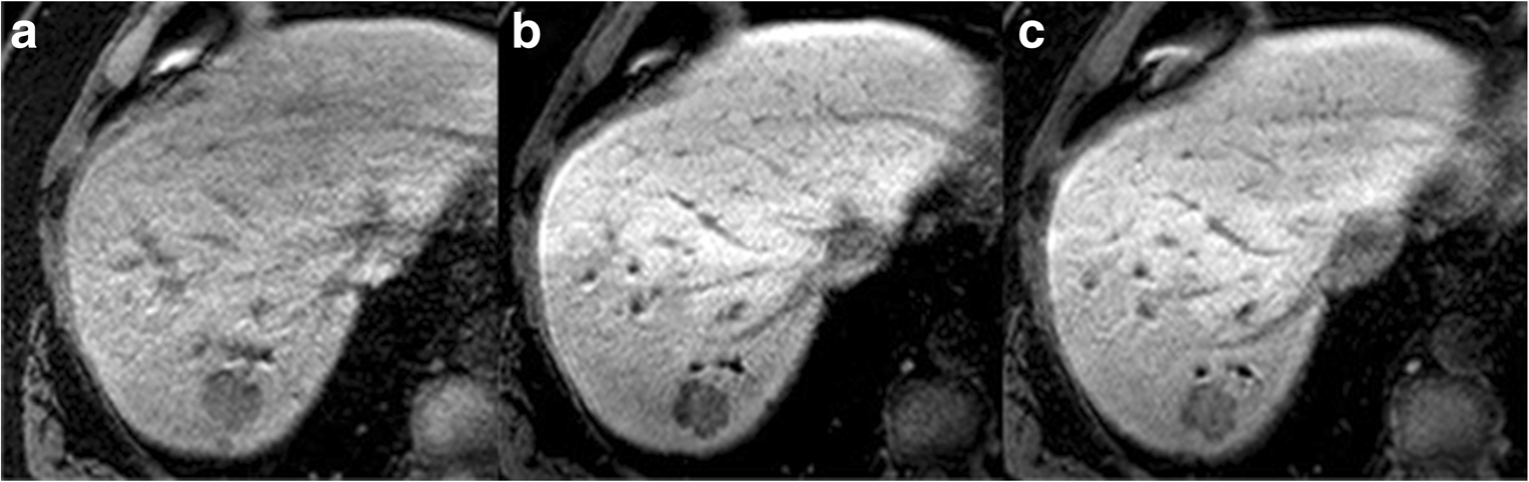
Pathology-confirmed colorectal liver metastases on preoperative 3D Axial T1 MRI at (a) precontrast, (b) 10-min post-gadoxetate injection, and (c) 20-min post-gadoxetate injection in a 60-year-old male demonstrating strong tumour enhancement

**Fig. 2:**
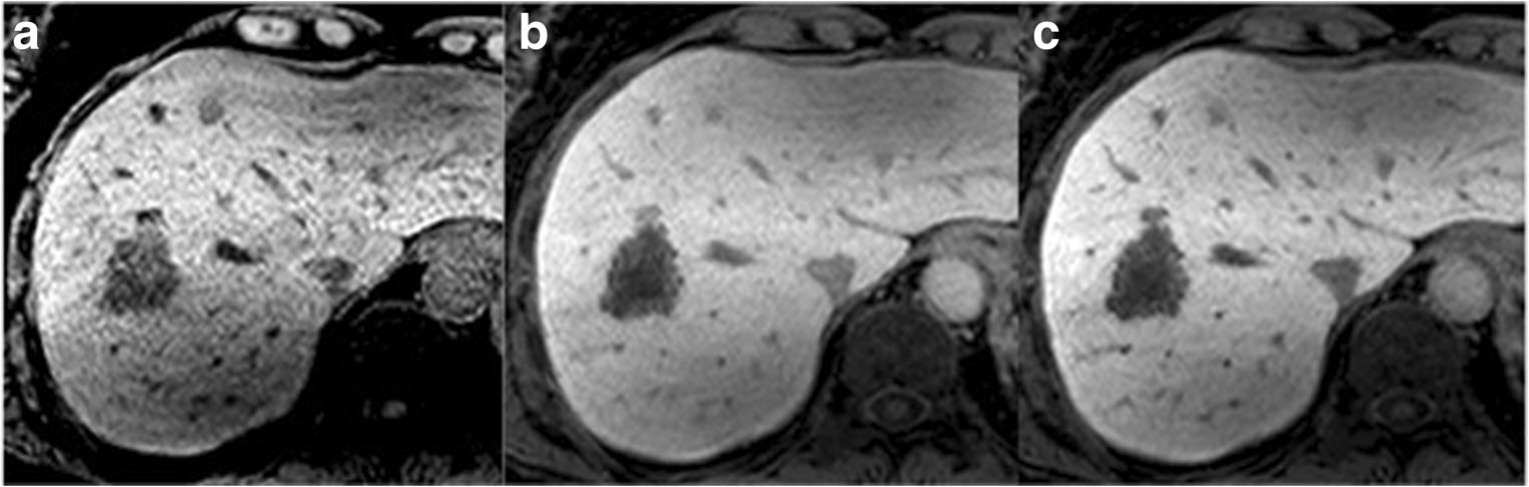
Pathology-confirmed colorectal liver metastases on preoperative 3D axial T1 MRI at (a) precontrast, (b) 10-min post-gadoxetate injection, and (c) 20-min post-gadoxetate injection in a 69-year-old male demonstrating weak tumour enhancement

Based on the Youden Index, the best threshold for predicting 3-year mortality using Target TuEn10 was 25%. Therefore, we dichotomised tumour enhancement into strong Target TuEn10 (≥ 25%) and weak Target TuEn10 (< 25%). Thirty-six patients (with 5 events) had strong Target TuEn10 and 29 (with 13 events) had weak Target TuEn10 (Figs. 1 and 2).

Patient with strong Target TuEn10 were more likely to have smaller tumours (*p* < 0.03) and metachronous metastases (*p* = 0.01). No other demographic data were significantly different between strong and weak Target TuEn10 groups (Table 2).

**Fig. 3:**
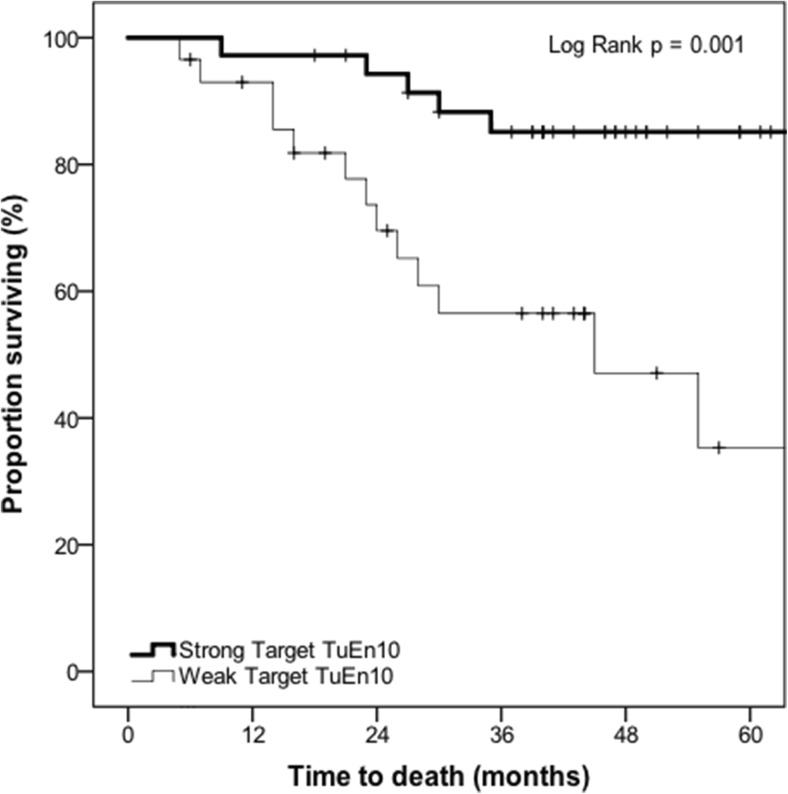
Kaplan-Meier survival curve demonstrating the association between target tumour enhancement at 10-min delayed phase (Target TuEn10) and overall survival in patients with colorectal liver metastases who had received preoperative gadoxetate-enhanced MRI prior to hepatectomy (*n* = 65) (*p* = 0.001)

Sixty-five patients had data available for Kaplan-Meier analysis. On Kaplan-Meier analysis, strong Target TuEn10 was associated with overall survival (log-rank *p* = 0.001) (Fig. 3). At 3 years (36 months), the proportion surviving was 85.1% (SE: 6.2%) (number of events = 5; number at risk = 27) for those with strong Target TuEn10 compared with 56.5% (SE: 10.0%) (number of events = 11; number at risk = 13) for those with weak Target TuEn10.

Sixty-three had data available for Cox regression analysis, in which strong Target TuEn10 was associated with overall survival (*p* = 0.009), independent of clinical risk score. The adjusted hazard ratio of Target TuEn10 and clinical risk score for overall survival were 0.24 (95% CI: 0.08-0.70) and 2.61 (95% CI: 0.98-6.98) respectively (Table 3).

**Table 3:** Cox regression model for adjusted hazard ratios for the association of target tumour enhancement at 10-min delayed phase imaging (Target TuEn10) on gadoxetate-enhanced MRI and overall survival (*n* = 63)

	Adjusted hazard ratio (95% confidence interval)	*p* value
Target TuEn10		
Weak	Reference	*p* = 0.009**
Strong	0.24 (0.08-0.70)	
Clinical risk score		
< 3	Reference	*p* = 0.055
≥ 3	2.61 (0.98-6.98)	

**p < 0.01

Post-hoc sensitivity analyses were performed for synchronous vs. metachronous metastases. When tumour size and synchronous metastases were included in the Cox regression model, Target TuEn10 had an adjusted hazard ratio of 0.23 (95% CI: 0.07-0.72).

### Survival analysis for target TuEn20

Based on the Youden Index, the best threshold for predicting 3-year mortality using Target TuEn20 was 77%. Therefore, we dichotomised tumour enhancement into strong Target TuEn20 (≥ 77%) and weak Target TuEn20 (< 77%). Forty-four patients (with 8 events) had strong Target TuEn20 and 20 patients (with 10 events) had weak Target TuEn20 (Figs. 1 and 2).

No demographic data were significantly different between strong and weak Target TuEn20 groups (Table 2).

**Fig. 4:**
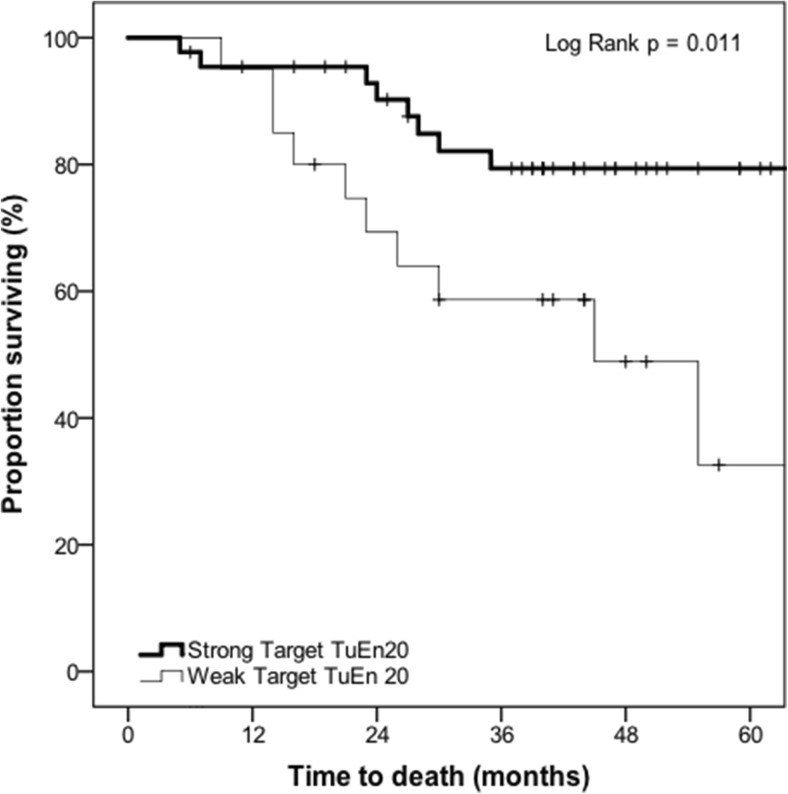
Kaplan-Meier survival curve demonstrating the association between target tumour enhancement at 20-min delayed phase (Target TuEn20) and overall survival in patients with colorectal liver metastases who had received preoperative gadoxetate-enhanced MRI prior to hepatectomy (*n* = 64) (*p* = 0.011)

Sixty-four patients had data available for Kaplan-Meier analysis, in which strong Target TuEn20 was associated with overall survival (log-rank *p* = 0.011) (Fig. 4). At 3 years (36 months), the proportion surviving was 79.4% (SE: 6.5%) (number of events = 8; number at risk = 29) for those with strong Target TuEn20 compared with 58.7% (SE: 11.2%) (number of events = 8; number at risk = 10) for those with weak Target TuEn20.

Sixty-two 62 patients had data available for Cox regression analysis, in which strong Target TuEn20 was associated with overall survival (*p* = 0.018), independent of clinical risk score. The adjusted hazard ratio of Target TuEn20 and clinical risk score for overall survival were 0.32 (95% CI: 0.12-0.82) and 3.20 (95% CI: 1.21-8.45) respectively (Table 4).

**Table 4:** Cox regression model for adjusted hazard ratios (HRs) for the association of target tumour enhancement at 20-min delayed phase imaging (Target TuEn20) on gadoxetate-enhanced MRI and overall survival (*n* = 62)

	Adjusted hazard ratio (95% confidence interval)	*p* value
Low TuEn 20		
Weak	Reference	*p* = 0.018*
Strong	0.32 (0.12-0.82)	
Clinical risk score		
< 3	Reference	*p* = 0.019*
≥ 3	3.20 (1.21-8.45)	

*p < 0.05

## Discussion

This study provides preliminary evidence that tumour enhancement of CRCLM on preoperative gadoxetate-enhanced MRI is associated with survival post-hepatectomy, after adjusting for known confounders. In patients who had strong target tumour enhancement on 10-min delayed phase, the proportion surviving at 36 months was 85.1% compared with 56.5% in patients who had weak target tumour enhancement, an absolute difference in overall survival of 28.6%. The adjusted hazard ratio, after considering known confounders, was 0.24. In patients who had strong target tumour enhancement on 20-min delayed phase, the proportion surviving at 36 months was 79.4% compared with 58.7% in patients who had weak target tumour enhancement, an absolute difference in overall survival of 20.7%. The adjusted hazard ratio, after taking into account known confounders, was 0.32. Therefore, tumour enhancement on delayed phase gadoxetate-enhanced MRI may represent a prognostic imaging biomarker of survival in this patient population.

Prior studies with extracellular contrast agents have demonstrated that late gadolinium enhancement is associated with tumour fibrosis, which is a known predictor of good outcomes post-hepatectomy in patients with CRCLM [4] . Future prospective, high-resolution, matched radiological-pathological correlation studies are required to determine whether this is also the case with gadoxetate. Prior studies with hepatobiliary-specific contrast agents have suggested that late gadolinium enhancement may be related to OATP1B3 expression and treatment response in a chemotherapy-naïve population [13, 14].

All patients were surgical candidates who received preoperative chemotherapy as per institutional clinical guidelines. However, in this retrospective cohort, the exact amount and type of chemotherapy varied between patients. This could affect outcomes and the results of this study. Further prospective studies controlling for chemotherapy regimens are required.

Although there is currently no literature on the timing of delayed phase imaging and correlation with tumour fibrosis in CRCLM, prior studies in the cardiac literature demonstrated that late gadolinium enhancement is best correlated with tissue fibrosis between 10-30 min using extracellular contrast agents [10]. In our study, tumour enhancement was correlated with overall survival at both 10- and 20-min delayed phases. Due to the small sample size, we were unable to directly compare tumour enhancement at 10- versus 20-min delayed phases and it is unclear based on our study if one time is better than the other or if both time points are required for evaluation of tumour enhancement. In this study, we specifically correlated delayed phase tumour enhancement with outcomes based on the prior literature [4]; however, it is possible that tumour enhancement at other phases (e.g. arterial, portovenous, or 2-min delayed phases) may provide additional prognostic information. This could be explored in future studies.

The ability to assess multiple tumours may be one of the significant advantages of imaging biomarkers over pathology and molecular biomarkers. There is growing evidence that tissue sampling is prone to error due to tumour heterogeneity among different tumours and even within the same tumour [15–17]. In heterogeneous tumour clones, sampling of tumours may not accurately measure the most aggressive tumour, which may be most likely to drive patient survival [18]. Additionally, pathology and molecular prognostic biomarkers cannot be easily assessed preoperatively. Evaluation of pathology requires tissue specimens, which may be difficult to obtain. Liver biopsy is often not feasible because of technical difficulty as well as the risk of needle tract seeding [19] . The proposed MRI biomarker addresses some of these limitations, since it is a cross-sectional technique that can identify and evaluate all tumours and, unlike current pathology or molecular biology techniques, it does not require tumour sampling.

Prognostic biomarkers may be particularly important in CRCLM because of the heterogeneity in outcomes in this patient population. It is increasingly understood that patients who have “good” biology may respond well to surgery even with features that were traditionally considered contraindications such as R1 resection or extrahepatic disease [19]. However, it is less clear how to determine preoperatively which patients have “good” biology and will benefit from treatment. Risk-stratification using prognostic biomarkers may be the key to addressing this challenge.

In our study, tumour enhancement at 10-min delayed phase was also associated with smaller tumour size and metachronous metastases. This may be because tumour enhancement is a good prognostic variable and was therefore associated with other known good prognostic variables [11]. In our post-hoc sensitivity analysis, tumour enhancement at 10-min delayed phase was associated with good outcomes independent of tumour size and the presence of metachronous metastases.

Gadoxetate-enhanced MRI is already routinely used in the preoperative diagnosis and staging of patients undergoing surgery for CRCLM, and 10- and 20-min delayed phase imaging is routinely performed on clinical gadoxetate-enhanced MRI [7]. This technique is widely available and measuring tumour enhancement is straightforward, does not require significant training, and can be done on standard clinical PACS software available at almost all cancer institutions that offer MRI. Therefore, if this imaging biomarker can be externally validated in future studies, it may be easily adapted for clinical practice.

There are a number of limitations to this study. This study was a small, preliminary, retrospective study. Larger multicentre studies are required to replicate and validate the results. In addition, prospective studies are required to determine how this finding may be used clinically for patient management. Further studies are also required to optimise techniques for measuring MRI enhancement.

In conclusion, tumour enhancement on gadoxetate-enhanced MRI is associated with long-term survival in patients with colorectal liver metastases and may represent a biomarker of prognosis.

## Abbreviations

CEA: Carcinoembryonic antigen
CRCLM: Colorectal liver metastases
MRI: Magnetic resonance imaging
PACS: Picture archiving and communication system
SNR: Signal-to-noise ratio
SNR0: Signal-to-noise ratio on precontrast phase
SNRX: Signal-to-noise ratio at “x” minutes post contrast
TuEn10: Tumour enhancement 10 min post contrast
TuEn20: Tumour enhancement 20 min post contrast
TuEnX: Tumour enhancement at “x” minutes post contrast
